# High-Precision Waveform Stacking Location Method for Microseismic Events Based on S-Transform

**DOI:** 10.3390/s25226965

**Published:** 2025-11-14

**Authors:** Hongpeng Zhao, Jiulong Cheng, Grzegorz Lizurek, Chuanpeng Wang, Yan Li, Dengke He, Zhongzhong Xu

**Affiliations:** 1 College of Earth Science and Surveying Engineering, China University of Mining and Technology-Beijing, Beijing 100083, China; zhao.hp.cumtb@gmail.com (H.Z.); he_dengke@cumtb.edu.cn (D.H.); xzzcumtb@163.com (Z.X.); 2 State Key Laboratory for Fine Exploration and Intelligent Development of Coal Resources, China University of Mining and Technology-Beijing, Beijing 100083, China; 3 Institute of Geophysics, Polish Academy of Sciences, 01-452 Warsaw, Poland; lizurek@igf.edu.pl; 4 CCTEG Coal Mining Research Institute, Beijing 100013, China; w13456728@163.com (C.W.);; 5 Tiandi Science and Technology Co., Ltd., Beijing 100013, China

**Keywords:** geotechnical engineering, microseismic source localization, Stockwell transform, characteristic functions, frequency and time coefficients

## Abstract

The waveform stacking location method achieves microseismic source localization by computing characteristic functions (CFs) and stacking multi-channel data, without phase picking. It has been widely applied in geotechnical engineering. However, the low signal-to-noise ratio (SNR) caused by weak event energy and ambient noise often degrades localization accuracy. To enhance the localization precision and stability under low SNR conditions, this study employs the Stockwell transform (S-transform) to convert noisy time-domain data into the time–frequency domain. By analyzing the energy distribution of microseismic signal and noise in the time–frequency domain, frequency and time coefficients are introduced to enhance the energy of microseismic signal. Event location is achieved through the computation of CFs and multiple-cross-correlation stacking. Comparison of the location results when computing the CFs by the new method, the short-term average to long-term average ratio (STA/LTA) method, and the envelope (Env) method under varying noise levels demonstrates the superior noise resistance and improved localization accuracy of the new method. Finally, the effectiveness of the new method is validated using real seismic data collected from a coal mine.

## 1. Introduction

In coal mining, tunnel excavation and other geotechnical engineering fields, the implementation of the project often induces rock fractures, generating microseismic signals. Microseismic monitoring technology enables real-time acquisition of rock fracture information and provides efficient early warning of disasters. The fundamental principle of microseismic monitoring technology involves collecting seismic wave signals generated by rock fracture via sensors. By further processing and analyzing these seismic wave signals, key parameters such as the location, magnitude, energy, and moment tensor can be determined. Source localization constitutes a critical component of microseismic monitoring, serving as the primary task for obtaining insights of rock mass instability and failure, which is essential for disaster early warning [1,2,3].

Traditional location methods are based on travel time. The Geiger method [4] proposed in the early 20th century iteratively solves equations to estimate source locations. Many scholars have made improvements to address the limitations in the Geiger algorithm’s iterative process. These improvements mainly include optimizing algorithms such as damped least squares [5], conjugate gradient method [6], and simplex location algorithm [7]. There are also regularization algorithms that combined centralization method and row balance pretreatment [8], as well as reverse travel time location method [9]. In these methods, phase identification and first break picking are required. Manual picking is costly, while automatic picking has poor performance. The results of automatic picking are unreliable in the situations with low signal-to-noise ratio (SNR) and overlapping waveforms [10]. The precision of first break picking is crucial for location results [11]. Furthermore, machine learning has also been utilized for seismic source localization [12,13], but its application in real engineering for source localization still faces challenges [14]. The primary challenge lies in data scarcity and imbalance. Without rigorous quality control, machine learning methods may capture spurious patterns associated with data inconsistencies rather than genuine geophysical signals, thereby compromising its generalizability and interpretability. The idea of migration stacking in reflection seismology has been adapted. They proposed some source location methods that do not require first break picking and use multi-channel waveform information [15,16]. These waveform stacking methods generally fall into two categories: Kirchhoff migration and interferometric migration [17]. The Kirchhoff migration method stacks waveforms from various sensors along the differential travel times [18,19]. In contrast, interferometric migration uses differential travel times between sensor pairs for common events, stacking cross-correlation functions to achieve event location [20]. Unlike Kirchhoff migration stacking, cross-correlation operations in interferometric migration consider the correlation among sensors when stacking along the differential travel times [21].

When performing the cross-correlation stacking method, the study area is often discretized into several grid points. For each grid point, characteristic functions (CFs) are calculated from original waveforms recorded by sensors, and then migrated and stacked according to the travel time difference to focus energy for source imaging. The calculation method of the CFs is crucial to the location results. Various CFs have been proposed to improve accuracy, including waveform envelope [22,23], semblance [24,25], short-term average to long-term average ratio (STA/LTA) [26,27,28], and kurtosis [29,30]. The introduction of the double-cross-correlation method [31] and multiple-cross-correlation [32,33,34] further enhanced correlation across multiple seismic channels. However, due to the weak energy of microseismic signals and non-stationary noise, current location methods remain challenged by noisy conditions. While denoising pre-processing is an option, it complicates the workflow and reduces the simplicity of waveform stacking. The time–frequency transform is a powerful tool for transforming microseismic records from the time domain to the time–frequency domain, revealing the complex structures of microseismic records in the time–frequency domain [35]. Researchers have successfully applied this method to various aspects of microseismic data processing, including signal denoising, first break picking, and seismic source localization [36,37,38]. Among various time–frequency techniques, the short-time Fourier transform (STFT) [39] employs a fixed window function, making it unsuitable for processing signals with multiple frequency bands. The wavelet transform can provide variable time–frequency resolution, but its resolution depends on the selected scale and mother wavelet function [40]. Although appropriate parameters can provide clear resolution, the selection of optimal parameters is challenging in itself. In contrast, the Stockwell transform (S-transform) adopts a Gaussian window whose width automatically adjusts with frequency [41], making it inherently adaptive. This inherent adaptability enables it to effectively extract time–frequency information [42], making it suitable for analyzing non-stationary seismic wave records with evolving frequency content over time. Our study employs the S-transform [43] to convert signals into the time–frequency domain. We analyze the energy distribution characteristics of both signals and noise in the time–frequency domain to calculate frequency and time coefficients. After enhancing the microseismic signal components and constructing the CFs, precise microseismic location under noisy conditions is then achieved through multiple-cross-correlation waveform stacking.

## 2. Methods

In geotechnical engineering, particularly in coal mining and tunnel excavation, construction activity induces rock fractures that generate microseismic signals. However, these signals are generally weak and easily contaminated by various noise [44], leading to a low SNR. Such noisy data poses a challenge for the cross-correlation stacking localization method, and the most critical step of this kind of method is the computation of the characteristic functions (CFs). A proper method for computing the CFs can significantly improve the algorithm’s performance. In the following sections, we use simulated noisy microseismic data to illustrate our method.

### 2.1. Construction of Characteristic Function

**Figure 1:**
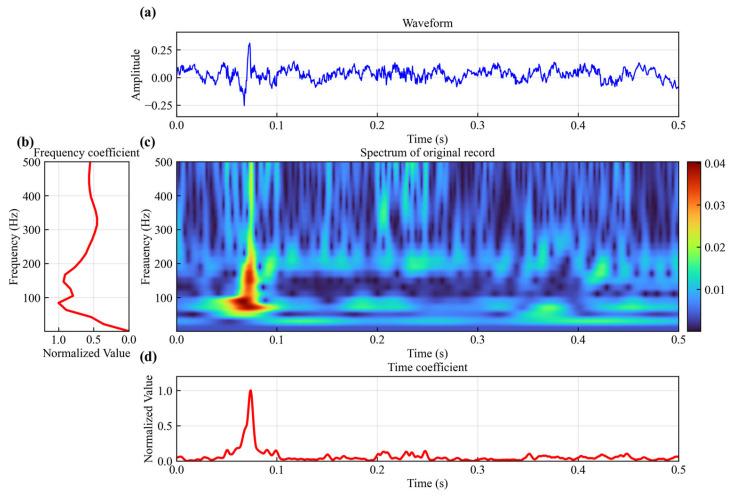
(**a**) Synthetic noisy data; (**b**) spectrum of synthetic noisy data; (**c**) frequency coefficients; (**d**) time coefficients.

Figure 1a shows noisy microseismic data. In the time domain, we can find that the amplitude of the microseismic signal is stronger than the noise. However, the microseismic signal is mixed with the noise, making it difficult to identify the first break. We use Formula (1) to perform S-transform on the synthetic waveform data w(t), converting the data from the time domain into a two dimensional function H(τ,f) that contains both time τ and frequency f. In order to speed up the calculation efficiency of S-transform, we use the 3-sigma rule to truncate the data outside the window in the calculation process, reducing unnecessary calculations.

(1) $$H(\tau ,f)=\int_{-\infty }^{+\infty }w(t)\frac{\left|f\right|}{\sqrt{2\pi }}e^{-\frac{{\left(\tau -t\right)}^{2}f^{2}}{2}}e^{-i2\pi ft}dt$$

Figure 1c shows the time–frequency spectrum of the noisy microseismic data. It can be observed that the intensity of the microseismic signal is greater than that of the noise. The spectrogram shows that the frequency of the microseismic signal is concentrated in the 80–200 Hz range, with energy primarily focused around 0.07 s. The noise can be categorized into two types: one overlaps with the microseismic signal in the same frequency band. The other type occupies different frequency bands from the microseismic signal, mainly distributed outside the 80–200 Hz range, spread in time from 0 to 0.5 s. The energy of both types of noise is dispersed, while the energy of the microseismic signal is focused and has a greater intensity than the noise.

In order to further highlight the microseismic signal from the noise signal, we propose to use the frequency coefficient and time coefficient to enhance the microseismic signal in two dimensions. Observing along the frequency dimension of the time–frequency spectrum, we can find that within the frequency band dominated by microseismic signals, the spectral energy changes abruptly over time, significantly exceeding that of energy changes in other frequency bands. To quantify this type of energy fluctuation, we use Equation (2) to calculate the spectral energy range p(f) corresponding to each frequency f in the absolute value of time–frequency spectrum H(τ,f).

(2) $$p(f)=\max\left[\left|H(\tau ,f)\right|\right]-\min\left[\left|H(\tau ,f)\right|\right]$$

The p(f) is the frequency coefficient. In Figure 1b, we can find that the values of p(f) within the frequency band of the microseismic signal are greater than other frequency bands.

From the time dimension of the time–frequency spectrum in Figure 1c, the energy within the time range corresponding to the microseismic signal is greater than that in other time ranges. The time coefficient h(τ) is obtained by integrating along the frequency dimension of $\left|H(\tau ,f)\right|$ and then squaring it.

(3) $$h(\tau )={\left[\int_{f_{1}}^{f_{2}}\left|H(\tau ,f)\right|df\right]}^{2}$$

Figure 1d shows that there is an obvious peak in the time coefficient h(τ) within the time span of the microseismic signal.

Enhance the part of microseismic signal in the time–frequency spectrum through the time coefficient h(τ) and frequency coefficient p(f) in both the time and frequency dimensions:

(4) $$H_{1}(\tau ,f)={\left[p(f)\right]}^{'}\cdot I_{1\times n_{\tau }}⊙\left|H(f,\tau )\right|⊙I_{n_{f}\times 1}\cdot h(\tau )$$

where $I_{n_{f}\times 1}$ is a column vector of length $n_{f}$, which is the number of rows in the time–frequency spectrum H(τ,f), and ⊙ is the Hadamard product of the matrix. $I_{n_{f}\times 1}$ is a column vector of length $n_{f}$, and $n_{\tau }$ is the number of columns in H(τ,f).

Integrate $H_{1}(f,\tau )$ along frequency to obtain the characteristic function CF(τ):

(5) $$CF(\tau )=\int_{-\infty }^{+\infty }H_{1}{\left(f,\tau \right)}^{2}df$$

**Figure 2:**
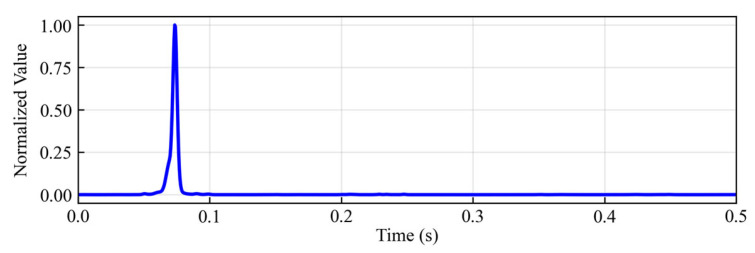
The characteristic function obtained by the new method.

As shown in Figure 2, the characteristic function CF(τ) exhibits a distinct peak around 0.07 s, successfully highlighting the microseismic signal from the noisy waveform data.

### 2.2. Multiple-Cross-Correlation Stacking Method

Based on reciprocity theory, the travel time $t_{x,i}$ from grid point x to sensor i equals the travel time $t_{i,x}$ from sensor i to grid point x. For propagation time calculation, we employ Fast Sweeping method (FSM) [45] for reverse propagation time computation. The FSM algorithm features high precision and rapid computation, effectively mitigating low computational accuracy issues in structures with drastic velocity changes or non-smooth model parameters, thereby yielding more stable results. Previous studies have demonstrated that the multiple-cross-correlation stacking method can significantly enhance high-resolution imaging performance. We use Formula (6) to perform multiple-cross-correlation to obtain the stacking imaging function W(x).

(6) $$W(x)=\sum_{i=1}^{n}\sum_{j=i+1}^{n}CF_{i}(t)CF_{j}(t+\tau_{0})$$

$CF_{i}$ and $CF_{j}$ are the characteristic functions of sensors *i* and *j*, respectively, and $\tau_{0}=t_{i,x}-t_{j,x}$.

## 3. Results

### 3.1. Application on Synthetic Model

**Figure 3:**
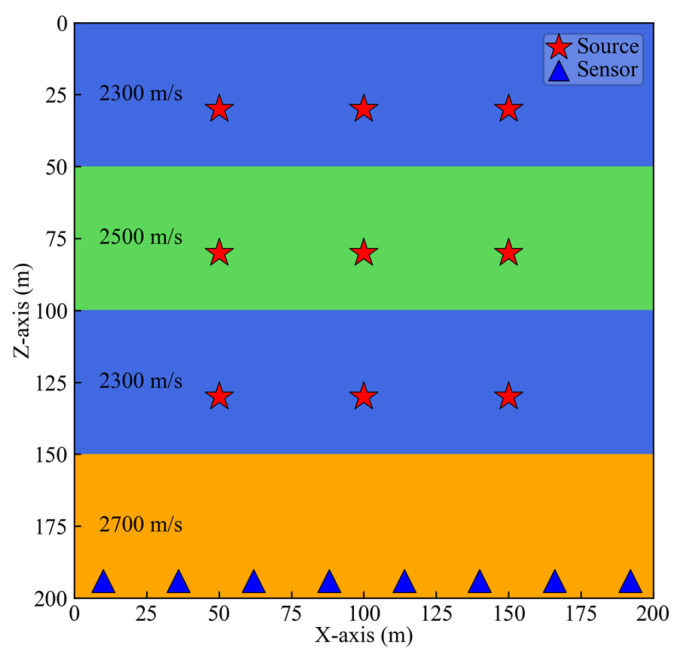
Four-layer model with low-velocity interlayer and locations of sources and sensors.

To validate the effectiveness of the new method, we constructed a four-layer model containing a low-velocity interlayer shown in Figure 3, with dimensions of 200 m × 200 m.

The study area was divided into 100 × 100 grids with a spacing of 2 m. Each layer had a thickness of 50 m, and the velocities of the four layers are 2300 m/s, 2500 m/s, 2300 m/s, and 2700 m/s, respectively.

**Figure 4:**
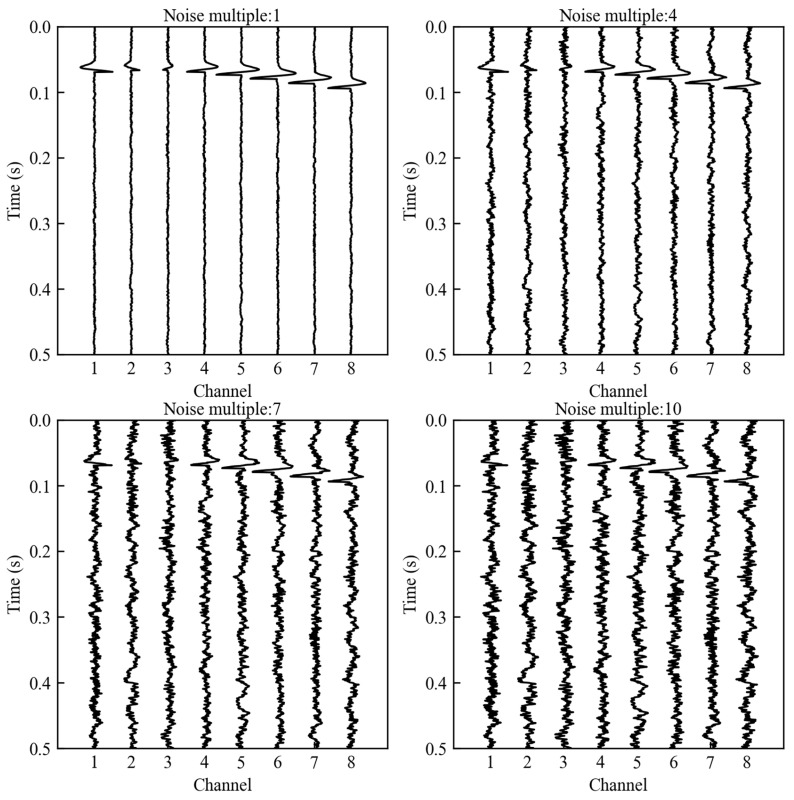
Noisy synthetic microseismic data with noise multiples of 1, 4, 7, and 10.

We placed nine microseismic sources across the first three layers. For each layer, three sources were set at the same depth. The Z-coordinates of the sources in the three layers were 50 m, 100 m, and 150 m, respectively. Each column of three source groups had the same X-coordinate, which was 30 m, 80 m, and 130 m, respectively. In the fourth layer, eight equally spaced seismic displacement sensors of identical depth were deployed. The source-time function was defined by a ricker wavelet with a dominant frequency of 50 Hz. A horizontal single force source was employed to excite the seismic source, ensuring that the Z-component data exhibits polarity reversal of the first motion. Then, we employed the finite difference method to simulate the wavefield generated by each source, extracting the P-wave component and adding non-stationary noise. The noise synthesis method followed the method described by Zeng [46], incorporating random noise, fixed frequency noise, and AM-FM noise. Ten progressively increasing multiples of noise were added to the synthetic signal. As shown in Figure 4, we present the data of sensors acquired at the source location X = 30 m, Z = 80 m. The figure displays signals with noise multiples of 1, 4, 7, and 10. It can be observed that as the noise level increases, the effective signal in Channels 2 and 3 becomes increasingly difficult to discern.

**Figure 5:**
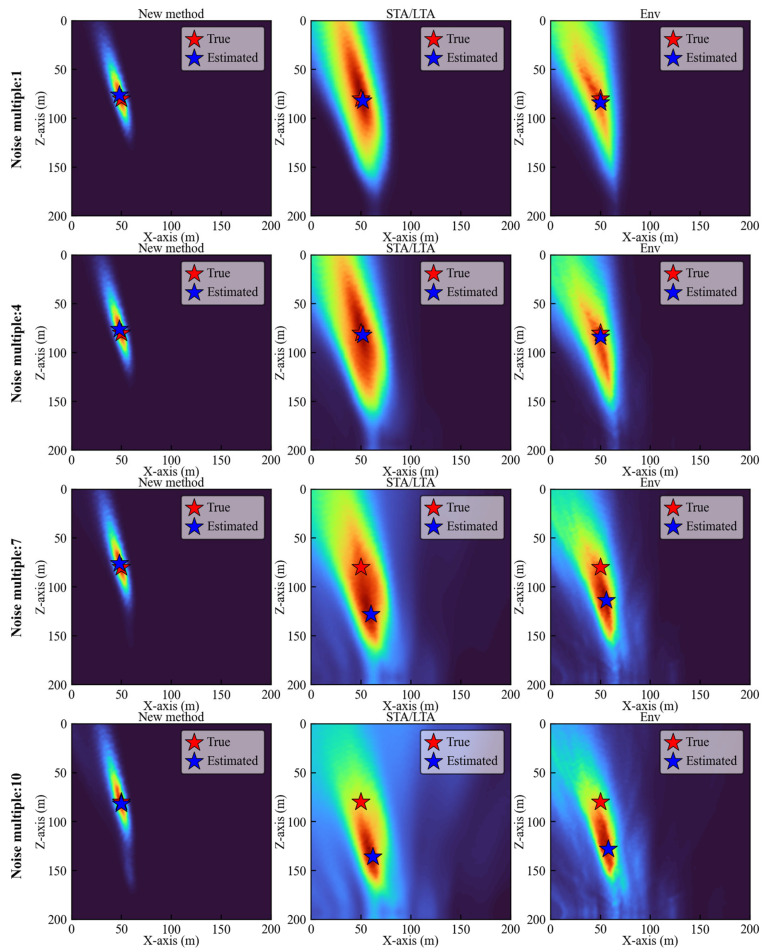
Location results of the new method, STA/LTA method and Env method for noisy synthetic microseismic data with noise multiples of 1, 4, 7, and 10.

For these four datasets, we compared the localization results when calculating the CFs by the new method, the STA/LTA method, and the Env method. As shown in Figure 5, at a 1× noise level, all three methods achieved satisfactory source localization performance. However, in terms of imaging quality, the new method produced significantly more focused results, with energy more concentrated near the source. As the noise level increases, both the STA/LTA and Env methods exhibit varying degrees of performance degradation, resulting in noticeable location inaccuracies. Particularly when multiples of noise reach 7 and 10, these methods produce significant errors along the *Z*-axis. Compared to the STA/LTA and Env methods, the new method consistently delivers the highest level of focusing performance in imaging results.

**Figure 6:**
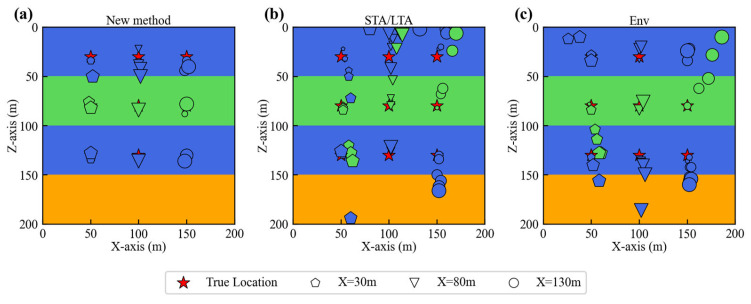
(**a**) Location results of the new method under noise-free conditions and 10 noise levels. (**b**) Location results of the STA/LTA method under none of noise and 10 noise levels. (**c**) Location results of the Env method under none of noise and 10 noise levels.

To further validate our conclusions, we performed source localization for nine sources under noise-free conditions and ten different noise levels. The localization results of the three methods are shown in Figure 6. The plotting color for each layer matches its respective layer, while different columns use different markers. The markers increase in size to represent the progression of noise from none to progressively higher levels. Figure 6a demonstrates that for the new method, all localization results cluster around the true source locations. Moreover, as noise levels increase, the localization deviation remains small. From the localization results of the two traditional methods in Figure 6b,c, the Env method demonstrates better performance to the STA/LTA method, exhibiting smaller localization errors. However, both traditional methods are significantly impacted by increasing noise levels, resulting in larger localization errors.

### 3.2. Error Statistics

**Figure 7:**
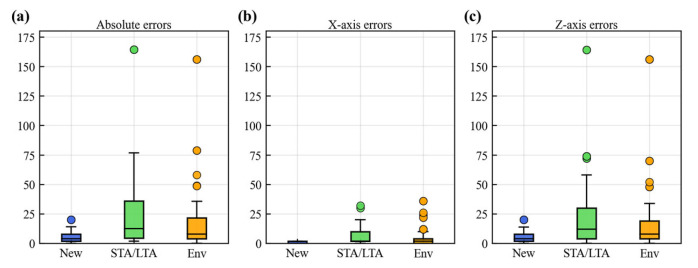
Location errors of the three methods. (**a**) Absolute errors; (**b**) *X*-axis errors; (**c**) *Z*-axis errors.

We used Formulas (7)–(9) to calculate the absolute error $E_{abs}$, *X*-axis error $E_{x}$, and *Z*-axis error $E_{z}$ of the positioning results of the three methods, and used box plots in Figure 7 to perform statistical analysis of the three errors.

(7) $$E_{abs}=\sqrt{{\left(x-x_{0}\right)}^{2}+{\left(z-z_{0}\right)}^{2}}$$

(8) $$E_{x}=\sqrt{{\left(x-x_{0}\right)}^{2}}$$

(9) $$E_{z}=\sqrt{{\left(z-z_{0}\right)}^{2}}$$

where (x,z) is the positioning result, and $\left(x_{0},z_{0}\right)$ is the true source location.

We perform location error analysis in Figure 7 using a box plot. The box represents the middle 50% of the data. Its upper boundary is defined by the upper quartile (the 75th percentile), and its lower boundary is defined by the lower quartile (the 25th percentile). The line inside the box indicates the median of the dataset. The difference between the upper quartile and the lower quartile gives the interquartile range (IQR). The upper whisker extends to the upper quartile plus 1.5 times the IQR, while the lower whisker extends to the lower quartile minus 1.5 times the IQR. The statistical outliers, which are data points lying below the lower whisker or above the upper whisker, are represented as individual dots in Figure 7.

In Figure 7, the results demonstrate that the new method exhibits lower errors across all three metrics compared to the other two methods, along with narrower error ranges, lower medians, and fewer outliers. In contrast, both the STA/LTA and the Env methods show higher median errors and greater variability relative to the new method, with a higher number of outliers. The STA/LTA method presents the widest error distribution, the highest median error, and most outliers, reaching maximum absolute and *Z*-axis errors of approximately 164 m, indicating the poorest overall accuracy and stability. The Env method performs slightly better than the STA/LTA method, yet its maximum absolute and *Z*-axis errors are still around 156 m.

It is worth noting that for both conventional methods, the location errors in the *X*-axis direction are smaller than those in the *Z*-axis direction. This discrepancy is likely attributable to the sensors which may provide weaker constraints in the vertical (*Z*-axis) direction. The new method, however, achieves markedly smaller location errors specifically in the *Z*-axis. In summary, the new method surpasses both the STA/LTA and Env methods in absolute error, *X*-axis error, and *Z*-axis error, demonstrating better location accuracy and stability. The smaller errors in the *Z*-axis particularly highlight the advantage of the new method, indicating stronger constraints in the vertical dimension.

**Figure 8:**
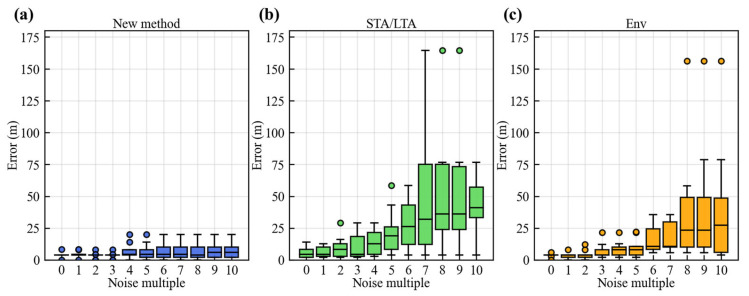
Variation in absolute location errors with increasing noise levels. (**a**) New method; (**b**) STA/LTA method; (**c**) Env method.

Figure 8 shows the absolute error performance of the new method, STA/LTA, and Env methods under different noise levels presented by box plot. The specific meanings of the elements in Figure 8 are the same as those in Figure 7. The box plots demonstrate that the new method maintains low and stable median errors with a narrow error range, indicating highly concentrated and tightly constrained localization results. The median errors of the new method remain consistently low, and even under high noise levels, the expansion of the error range is limited, reflecting strong noise resistance and precise location capability. In contrast, both the STA/LTA and Env methods exhibit increased median errors and error ranges as the noise level rises. Particularly under high noise conditions, their errors distribution become more dispersed. This highlights the better performance of the new method in noisy conditions.

### 3.3. Comparison of Time Cost

In this study, all three methods were employed to test nine seismic sources under noise-free conditions and across ten different noise levels. For each method, the calculation time of these 99 locations was averaged, and the average time cost of the 99 seismic source locations is shown in Table 1. All our codes were implemented in Python 3.8.19 and executed on a personal laptop equipped with an Intel^®^ Core™ i5-10300H CPU and 32 GB of RAM.

As indicated in Table 1, the new method required an average of 0.177 s per seismic source localization, while the STA/LTA and Env methods need 0.018 s and 0.020 s, respectively. The increase in computational time of the new method compared to the other two methods is attributed to the implementation of the S-transform, which is a relatively time-consuming process. However, the absolute time overhead of the new method is not substantial, as it only takes 0.177 s per localization event on a personal laptop. This duration is practically acceptable, enabling immediate data processing and yielding results promptly after the occurrence of microseismic events.

### 3.4. Non-Uniform Sensor Arrays

**Figure 9:**
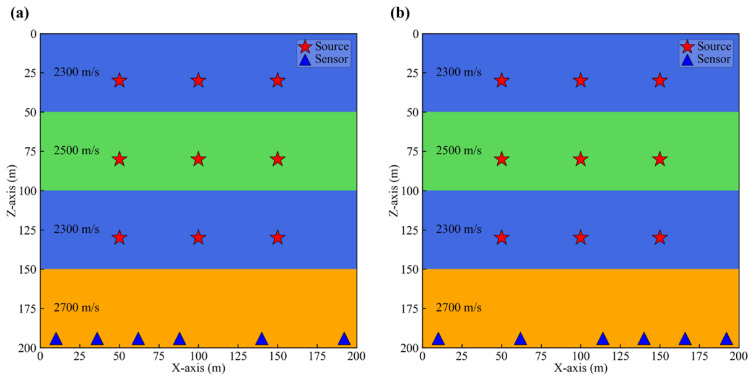
Non-uniform sensor array. (**a**) The sparse array on the right. (**b**) The sparse array on the left.

Sensor arrays are often deployed in a non-uniform manner due to site constraints. To evaluate the performance of new method under such non-uniform array configurations, we changed the sensor layout originally shown in Figure 3. Specifically, we removed the 5th and 7th sensors on the right side, and the 2nd and 4th sensors on the left side, obtaining in two distinct non-uniform array geometries illustrated in Figure 9a,b. In the configuration of Figure 9a, the right side of the array is sparse, while in Figure 9b, the left side exhibits sparsity.

Table 2 presents the average absolute location errors of the three methods under three arrays: the uniform sensor array in Figure 3 and the two non-uniform arrays (a and b) in Figure 9. Compared to the uniform array, all three methods exhibit increased location errors when using non-uniform sensor arrays. This is expected, as non-uniform arrays inherently provide weaker constraints, leading to a decline in location accuracy. It is noteworthy that our method still achieves the smallest errors even with non-uniform arrays. Furthermore, when using the right-sparse non-uniform Array a, both the new method and the Env method obtain smaller errors than with the left-sparse Array b. In contrast, the STA/LTA method performs better with Array b. This may be attributed to the random of noise. Nevertheless, under these variable conditions, our method consistently demonstrates best performance compared to the other two methods.

### 3.5. Velocity Error Analysis

Velocity errors are always present in practical microseismic event location and cannot be overlooked. To test the sensitivity of the three methods to velocity errors, we introduced velocity errors of ±5%, ±10%, and ±15% into the velocity model. The absolute errors between the locations determined by the three methods under the six velocity errors and the true source locations are presented in Table 3. The location results of all methods deviate from the true source locations. When the velocity model values are increased, the location results deviate further from the true location, and the location errors are larger than those when the velocity model values are decreased. When the true velocity differs from the model velocity, an error arises between the calculated travel time and the true travel time. This travel time error depends on the difference in slowness (the reciprocal of velocity). For the same absolute velocity error, an overestimated velocity leads to a larger difference in slowness, consequently causing a larger travel time error and a larger location error.

In nearly all velocity error experiments, the new method exhibited the smallest error, followed by the Env method, while the STA/LTA method showed the largest error. However, an exception occurred at a velocity error of −15%, where the error of the new method was 27.49 m, larger than the 26.67 m error of the Env method. This can be attributed to the new method’s higher time resolution, which allows it to capture phase and amplitude information in the seismic signal more accurately. Precisely this higher waveform resolution makes the new method more sensitive to errors induced by velocity underestimation.

### 3.6. Application on Real Microseismic Record

**Figure 10:**
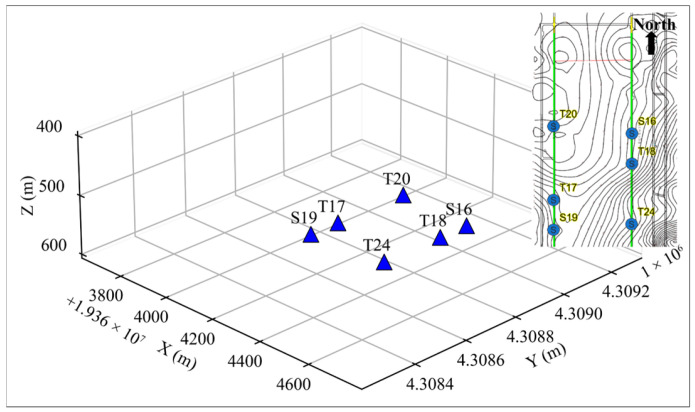
Map of monitoring sensors.

To verify the effectiveness of the new method, we used real mine seismic monitoring data. The real microseismic data were collected from a microseismic monitoring system deployed in a coal mine in Inner Mongolia. As shown in Figure 10, a total of six sensors were deployed in the roadways on both sides of the working face. Among them, T24 was a three-component sensor. The Z-component data recorded by the T24 sensor, along with data from the other five single-component (Z) sensors, were used in this study. These sensors operated at a sampling rate of 500 Hz, and the P-wave velocity is 3700 m/s.

**Figure 11:**
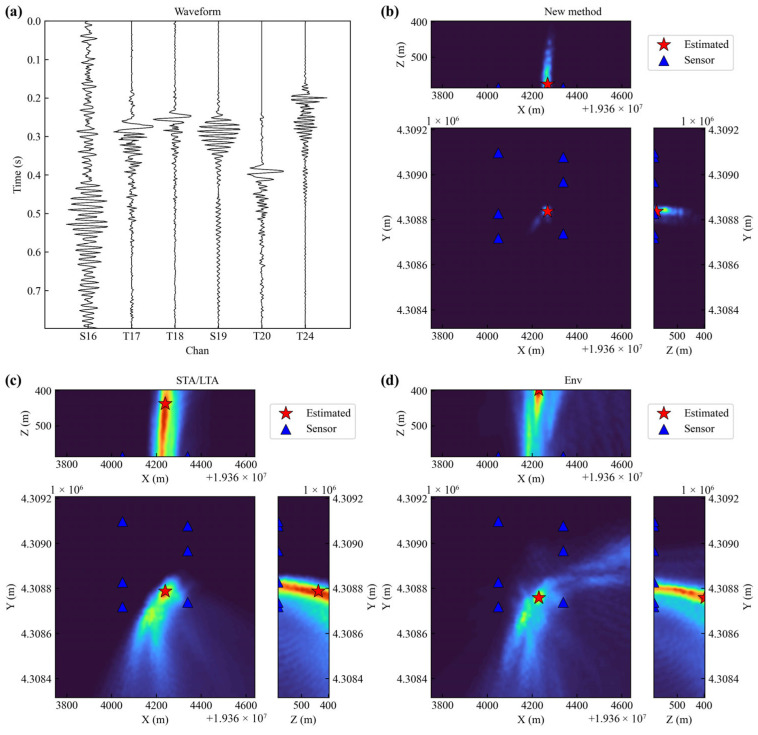
(**a**) Waveform of microseismic event. (**b**) Location result of new method. (**c**) Location result of STA/LTA method. (**d**) Location result of Env method.

Figure 11a displays the seismic wave data from a microseismic event recorded by the sensors. It can be observed from the data that the S16 sensor was affected by persistent noise, which was likely generated by the continuous operation of the coal mining machine. Due to spatial constraints and limited underground construction conditions, the number of deployed sensors is restricted. This makes the data from each sensor particularly crucial for microseismic source location. Consequently, the algorithm used must have a certain degree of noise resistance.

The spatial domain was discretized into grids with a 10 m interval. A comparative analysis of the localization performance of the three methods was conducted on a 90 × 90 × 20 grid. Figure 11b–d present the 3D localization results of the new method, STA/LTA, and Env methods for the microseismic event recorded in Figure 11a. The results demonstrate that the new method exhibits an advantage in spatial constraint, particularly along the *Z*-axis. It accurately confines the event location to a specific Z-level, with energy highly concentrated in source region, forming a distinct bright peak in the imaging result. In contrast, the Env method shows more dispersed localization results along the *Z*-axis, with energy distributed over a broader range. The STA/LTA method performs the poorest, displaying the weakest *Z*-axis constraint, and the widest energy distribution.

**Figure 12:**
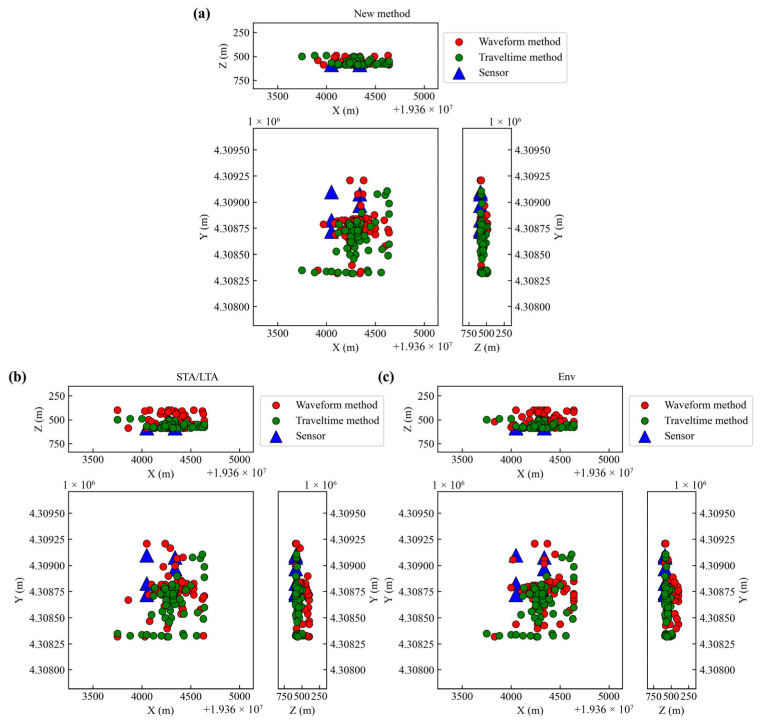
Microseismic dataset location results. (**a**) Location result of new method. (**b**) Location result of STA/LTA method. (**c**) Location result of Env method.

We conducted location tests on 80 microseismic events recorded by the system. The localization process used only the P-waves, with a grid spacing of 10 m and a grid size of 90 × 90 × 20. For comparison, we employed the reverse travel time location method [9] and compared its results with those of the three methods, as illustrated in Figure 12. The results of the new method show strong consistency with those of the travel time method. In the *Z*-axis direction, the localization results of the new method are highly concentrated within a range of approximately 100 m above the roadway, demonstrating better vertical constraint. In contrast, the *Z*-axis results of the STA/LTA and Env methods are more dispersed, exhibit a larger span, and show poor constraint, differing from the results of the travel time method. This indicates that the new method achieves higher localization accuracy and stability in the *Z*-axis direction, enabling more precise constraint of the event location in practical applications.

## 4. Conclusions

The novel method proposed in this study demonstrates significant advantages in microseismic source localization, particularly in noisy environments. By applying the S-Transform to convert signals into the time–frequency domain, it adaptively enhances microseismic signals using frequency and time coefficients, constructs a characteristic function, and performs multi-channel stacking to achieve high-precision source imaging. The localization results maintain high accuracy and stability even under low SNR conditions. This is especially critical in geotechnical engineering settings, where noise generated by mechanical equipment (e.g., coal-cutting machinery) often overlaps with the frequency band of microseismic signals. Validation using simulated data shows that the new method maintains superior localization accuracy across varying noise levels, exhibiting a narrower error range, lower median error, and higher spatial resolution compared to traditional methods such as STA/LTA and Env. Practical data tests further confirm the method’s strong constraint capability in the vertical direction, which is crucial for applications like mining safety, as accurate depth estimation directly determines the risk assessment of rock fracturing. The new method concentrates signal energy within a narrower time window. This enhanced time focusing implies that accurate migration imaging can only be achieved within a more precise (shorter) time range. A shorter time window corresponds to a more compact spatial domain. Consequently, when this method is applied, the seismic source is constrained to a smaller spatial volume, resulting in a stronger constraint along the *Z*-axis. Furthermore, since the method confines the source to a smaller spatial region, the constraint along the *X*-axis or on the X-Y plane is also improved. However, this advantage is less pronounced in practice because the sensors typically provide strong constraints along the *X*-axis or X-Y plane due to their wide coverage.

By integrating time–frequency analysis with waveform superposition, this method advances microseismic localization technology, provides new insights for the progression of microseismic monitoring, and demonstrates considerable potential and algorithmic robustness. It also offers a new tool for hazard early warning in geotechnical engineering. However, it must be acknowledged that the S-transform introduces additional computational complexity, which slows down the algorithm. However, this slowness is relative to faster methods and does not imply that the computation time itself is particularly slow. As shown in Table 1, the time required for a single source localization is only 0.177 s, which is entirely acceptable. This duration is sufficient to meet the demands of real-time monitoring. All computations in this study were performed on a personal laptop with an Intel^®^ Core™ i5-10300H CPU and 32 GB of RAM. Even when dealing with larger-scale sensor arrays in the future, the method can be readily extended, since its processing time does not increase exponentially but rather linearly with the number of sensors. The total processing time would essentially be the base time required to process a single sensor multiplied by the number of sensors. Furthermore, the data processing in this study was conducted using a serial computation approach. But these processing operations are independent of each other; they can be fully parallelized. In real-time monitoring scenarios with larger sensor networks, a parallel computing strategy could be adopted to accelerate the computations. Moreover, while the current implementation uses a CPU for computation, employing GPU-based parallel computing in the future could significantly enhance the data processing capability of this method for large-scale sensor networks. In addition, the successful application of machine learning methods to geophysical problems provides us with new insights [47,48]. Future work may use machine learning techniques to further accelerate our location method.

## Figures and Tables

**Table 1:** Average time cost of seismic source location.

	New Method	STA/LTA	Env
Time (s)	0.177	0.018	0.020

**Table 2:** Comparison of average absolute location errors.

	New Method	STA/LTA	Env
Error of uniform array (m)	5.97	26.77	17.63
Error of Array a (m)	8.67	30.82	12.44
Error of Array b (m)	10.30	24.82	14.68

**Table 3:** Comparison of absolute location error with different velocity errors.

Method	+15%	+10%	+5%	−5%	−10%	−15%
Error of New (m)	31.82	24.60	13.92	9.04	17.76	27.49
Error of STA/LTA (m)	34.59	32.80	27.91	27.59	28.75	34.02
Error of Env (m)	35.03	25.20	19.81	19.63	23.91	26.67

## Data Availability

The synthetic data and corresponding codes used in this study have been uploaded to: https://github.com/Xiake26/location-method (accessed on 12 November 2025).
